# Frequency‐dependent transmission of *Batrachochytrium salamandrivorans* in eastern newts

**DOI:** 10.1111/tbed.14043

**Published:** 2021-03-09

**Authors:** Adrianna Tompros, Andrew D. Dean, Andy Fenton, Mark Q. Wilber, Edward Davis Carter, Matthew J. Gray

**Affiliations:** ^1^ Center for Wildlife Health Department of Forestry, Wildlife, and Fisheries University of Tennessee Institute of Agriculture Knoxville TN USA; ^2^ Institute of Infection, Veterinary and Ecological Sciences University of Liverpool Liverpool UK; ^3^ Department of Ecology, Evolution and Marine Biology University of California‐Santa Barbara Santa Barbara CA USA

**Keywords:** amphibian, *Batrachochytrium*, density‐dependent transmission, disease, fungus, model

## Abstract

Transmission is the fundamental process whereby pathogens infect their hosts and spread through populations, and can be characterized using mathematical functions. The functional form of transmission for emerging pathogens can determine pathogen impacts on host populations and can inform the efficacy of disease management strategies. By directly measuring transmission between infected and susceptible adult eastern newts (*Notophthalmus viridescens*) in aquatic mesocosms, we identified the most plausible transmission function for the emerging amphibian fungal pathogen *Batrachochytrium salamandrivorans* (*Bsal*). Although we considered a range of possible transmission functions, we found that *Bsal* transmission was best explained by pure frequency dependence. We observed that >90% of susceptible newts became infected within 17 days post‐exposure to an infected newt across a range of host densities and initial infection prevalence treatments. Under these conditions, we estimated *R_0_ = 4.9* for *Bsal* in an eastern newt population. Our results suggest that *Bsal* has the capability of driving eastern newt populations to extinction and that managing host density may not be an effective management strategy. Intervention strategies that prevent *Bsal* introduction or increase host resistance or tolerance to infection may be more effective. Our results add to the growing empirical evidence that transmission of wildlife pathogens can saturate and be functionally frequency‐dependent.

## 1 INTRODUCTION

Emerging infectious diseases can have significant impacts on biodiversity, ecosystem processes and human life (Buck & Ripple, 2017; Morens & Fauci, 2013; Scheele et al., 2019). Recent emergences of *Batrachochytrium dendrobatidis* (*Bd*), *Pseudogymnoascus destructans* and SARS‐CoV‐2 are evidence that wildlife pathogens and zoonoses can have irreversible effects on susceptible host populations and cost global economies trillions of dollars to mitigate (Alves et al., 2014; Fauci et al., 2020; Frick et al., 2010; Heymann & Shindo, 2020; Lips, 2016; Scheele et al., 2019). A fundamental step to disease mitigation is understanding pathogen transmission, which typically includes host contact and environment pathways (Begon et al., 2002; Lange et al., 2016; Langwig et al., 2015; Loh et al., 2015). Epidemiological models can play an important role in elucidating dominant transmission pathways, which can inform disease management strategies (Lange et al., 2016; McCallum, 2016; Woodhams et al., 2011). Disease management can range from modifying host densities and contact rates to treating individuals with vaccines or therapeutics that increase resistance or tolerance to infection (Woodhams et al., 2011). Hence, identifying the functional form of pathogen transmission in novel disease systems is an important first step in disease management (Orlofske et al., 2018).

Density‐ and frequency‐dependent transmission are functional forms common to various wildlife disease systems (Devenish‐Nelson et al., 2014; Orlofske et al., 2018). Density‐dependent transmission describes a system in which the number of host contacts increases linearly with population density (McCallum et al., 2001), whereas in a frequency‐dependent system the host contact rate is constant irrespective of population density; hence, infection depends only on the frequency of infected individuals (Begon et al., 2002; McCallum et al., 2001). In many disease systems, these functions may not adequately capture transmission (Hopkins et al., 2020). Rather, a more appropriate functional form for transmission often transitions between these two extremes, such as density dependence occurring at low host densities and frequency dependence at high densities (i.e. Holling's type II; McCallum et al. 2001; McCallum et al. 2017; Orlofske et al., 2018). Non‐linear transmission has been described in several wildlife disease systems, such as cowpox virus in rodents and brucellosis in elk (Cross et al., 2013; Smith et al., 2009). Given the potential natural variation in pathogen transmission, testing and fitting various transmission functions for novel pathogens are a proactive strategy to elucidating influential transmission pathways and identifying successful disease management strategies.

The emerging chytrid fungus, *Batrachochytrium salamandrivorans* (*Bsal*), is an invasive amphibian pathogen believed to be from Asia that is currently spreading across Europe and causing mass mortality events of several *Salamandridae* species (Lötters et al., 2020; Martel et al., 2014, 2020). *Bsal* creates necrotic ulcerations through the skin of amphibians (Van Rooij et al., 2015), which likely affects osmoregulation and creates opportunities for secondary bacterial infection. *Bsal* is commonly found in the European amphibian pet trade (Nguyen et al., 2017; Sabino‐Pinto et al., 2018), where it has caused economic losses (Fitzpatrick et al., 2018), and is believed to be the pathway for spillover events to wild amphibian populations (Martel et al., 2014). Currently, *Bsal* has yet to be detected in North America (Klocke et al., 2017; Waddle et al., 2020), but its projected impacts show that its introduction could be devastating to biodiversity (Yap et al., 2015). The introduction of *Bsal* to the United States, specifically the southeastern region, could decimate amphibian populations and cause the extinction of endemic and rare salamander species (Carter et al., 2020; Richgels et al., 2016).

Some models of *Bsal* epidemiology have been developed using European fire salamanders (*Salamandra salamandra*) and eastern newts (*Notophthalmus viridescens*) as the host species (Canessa et al., 2018; Islam et al., 2021; Malagon et al., 2020; Schmidt et al., 2017). However, these models did not explicitly measure transmission; they assumed functional forms *a priori* ranging from density‐ and frequency‐dependent to non‐linear. No studies have yet measured *Bsal* transmission under experimental conditions and compared candidate models. Under experimental conditions, population attributes that impact transmission, such as density and initial infection prevalence, can be manipulated to compare transmission functions (Hopkins et al., 2020). The aim of our study was to identify the functional response for *Bsal* transmission in a host species (*N. viridescens*) that is widely distributed in North America (Petranka, 2010). Given that contact rates of uninfected eastern newts appear to be non‐linear (Malagon et al., 2020), we hypothesized that transmission would follow Holling's type II functional response.

## 2 METHODS

### 2.1 Model organism

Adult eastern newts were collected in Tennessee, USA, from Knox County (35.846639, −83.872116), Anderson County (36.045596, −84.193765) and Catoosa Wildlife Management Area (36.050606, −84.802038; 36.061868, −84.804617) in September 2019 under Tennessee Wildlife Resources Agency Science Collection Permit #1504. Newts were transported by vehicle to the Johnson Animal Research and Teaching Unit at the University of Tennessee in <3 hr and group‐housed (ca. 15 newts per 6‐L container) at room temperature (20–22°C) for approximately 20 days prior to heat treatment (discussed below). All research described herein was conducted in a biosecure animal research facility and was approved by University of Tennessee Institutional Animal Care and Use Committee Protocol #2623.

### 2.2 Experimental design

Because co‐infections with *Bd* can affect *Bsal* pathogenicity in eastern newts (Longo et al., 2019), all newts (*n* = 290) were heat‐treated in an environmental chamber for 10 days at 30°C to eliminate possible *Bd* infections (Bletz, 2013). After 10 days, the temperature was lowered 2°C per day to 14°C before placing newts into the experimental tanks. Newts were uniquely marked via toe clipping and randomly assigned to 20 circular, 1‐m^2^ tanks. Tanks were connected to a flow‐through, dechlorinating water system and heat‐chilling unit that maintained depth at 30 cm and temperature at 14°C—a temperature that previous research showed *Bsal* transmission was possible in eastern newts (Malagon et al., 2020). Water flow into tanks was approximately 20 L per hour; hence, water in tanks turned over approximately 2 times per day. Every three days, newts were fed frozen bloodworms and tanks were scooped with nets to remove waste and other organic debris.

We used eastern newt density treatments (2–8 newts per m^2^) that are typically found in the wild (Malagon et al., 2020). In addition, because previous work reported that newt contact rates followed Holling's type II functional response (Malagon et al., 2020), we included several high newt densities (10–32 newts per m^2^, Table 1) in order to evaluate whether overall transmission saturated with increasing density. We crossed each host density with 1–3 initial infection prevalence treatments (12.5%, 25%, 50%) to evaluate how the force of infection was impacted by change in prevalence (Greer et al., 2008). This range of initial infection prevalence treatments was typical for amphibian studies modelling pathogen transmission (Greer et al., 2008; Rachowicz & Briggs, 2007). This design sought to maximize the overall range of density and prevalence combinations for subsequent model fitting (see below), rather than to maximize replication at a more limited number of combinations.

**TABLE 1:** Density of adult eastern newts (*Notophthalmus viridescens*) and initial *Bsal* infection prevalence in 20 1‐m^2^ aquatic mesocosms

Total density	Initial infection prevalence^a^		
	12.5%	25%	50%
2			1
4		1	2
6			3
8	1	2	4
10			5
12		3	6
14			7
16	2	4	8
18			9
20		5	10
32	4	8	16

^a^ Table interior shows the number of infected newts at the start of the experiment.

Newts used to initiate the epidemic in each tank were randomly selected and exposed to a high dose of *Bsal* zoospores (2.56 × 10^6^ zoospores/ml) in 9 ml of autoclaved dechlorinated water and 1 ml of inoculum for 24 hr (Malagon et al., 2020), which is approximately 6 times the infectious dose (ID)‐50 for eastern newts (M. J. Gray and E. D. Carter, unpubl. data). *Bsal* cultures were grown on TGhL agar plates, and each plate was flooded with 6 ml of autoclaved dechlorinated water to harvest zoospores (Carter et al., 2020). Zoospores were filtered through a 20‐µm filter to remove sporangia and were enumerated using a hemocytometer (Carter et al., 2020) and verified by flow cytometry.

After the 24‐hr exposure, infected newts were placed into tanks with susceptible newts corresponding to their randomly assigned infection prevalence and density treatment (Table 1). Every three days for up to 60 days, newts were removed from tanks using a clean net, identified and swabbed using a standardized protocol to detect *Bsal* infection status and infection intensity (i.e. load) on the skin (Blooi et al., 2013; Boyle et al., 2004). A different clean net was used for each newt, each individual was placed in a clean plastic bag, and gloves were changed between handling individuals to prevent cross‐contamination (Gray et al., 2018).

### 2.3 Infection status and load

All swabs were placed in a microcentrifuge tube labelled with the individual's identification number and swab date, and stored at −80°C until processed. To detect *Bsal* and estimate loads, genomic DNA was extracted from each swab using the QIAamp 96 DNA QIAcube HT Kit (Qiagen, Hilden, Germany), and qPCR was performed similar to Blooi et al. (2013) using the Applied Biosystems QuantStudio 6 Flex qPCR instrument (Thermo Fisher Scientific Inc.). All samples were run in duplicate and declared positive if both replicates reached cycle threshold prior to 50 amplification cycles (Carter et al., 2020).

To minimize the risk of false infection declarations, the following decision rules were used to determine the infection status of newts for the purpose of modelling transmission: (a) two or more consecutive swabs of the same infection status were considered true readings and left unchanged, (b) a single negative swab straddled by two positive swabs was considered a false negative and counted as a positive reading, and (c) once false negatives were accounted for, a single‐positive swab straddled by two negative swabs was considered a false positive and counted as a negative reading. Thus, for example, a sequence of the form NNPNPP was treated as NNPPPP (i.e. false negatives took precedence over false positives in ambiguous sequences). Hence, following these criteria, a newt was considered to have cleared a *Bsal* infection if the individual met one of the positive criteria followed by at least two consecutive negative swabs. Using the second criterion, we estimated the sensitivity of our qPCR method, which was 96.5%. Re‐infection was declared if a cleared individual became subsequently infected following the positive swabbing criteria. We also used qPCR to estimate pathogen load per treatment by averaging loads among individuals per experimental unit (i.e. mesocosm).

### 2.4 Statistical analyses: survival and pathogen load

We monitored survival twice daily for 62 days and removed infected individuals that died in <12 hr. We used Kaplan–Meier survival analyses to test for differences in mortality rate among host density and infection prevalence treatments. We used weighted least‐squares regression to test whether mean *Bsal* load in a mesocosm differed with host density or infection prevalence treatments. We weighted each mean log *Bsal* load estimate by the number of initially infected newts, such that load estimates from mesocosms with more infected individuals received higher weights in the analysis.

### 2.5 Modelling transmission

We used qPCR results from the first two swabs (days 2 and 5 post‐exposure) to parameterize our transmission model. As no individuals died nor cleared infection by day 5 post‐exposure, restricting our analysis to this time period allowed us to model transmission processes only; this corresponds to the assumption that death and recovery take place on a slower timescale than infection. Extending the methods of Greer et al. (2008), we directly calculated the probability distribution of susceptible individuals at each time step, rather than simply assuming a binomial distribution, and used a maximum‐likelihood approach to estimate the transmission parameters.

We defined $p_{S|N,S_{0}}\left(t\right)$ to be the probability of S susceptible individuals at time *t* in a population of size $S_{0}+I_{0}=N$, given that there were $S_{0}$ susceptible and $I_{0}$ infected individuals at t=0. Note, *S, I and N* were absolute numbers of individuals in the mesocosms. Neglecting death and recovery, the probability distribution of susceptible individuals was then determined by the system of $S_{0}+1$ differential equations,

$$\frac{dp_{S|N,S_{0}}}{dt}=\phi_{S+1,N}p_{S+1|N,S_{0}}‐\phi_{S,N}p_{S|N,S_{0}},\;\;\;\;\;\;\;\;\;\;0\leq S\leq S_{0},$$

where $\phi_{S,N}$ was the transmission function that defines the infection rate in a population of size N with S susceptible individuals. Note that as we assumed a fixed population size, the number of infected individuals was simply I=N‐S. Following Begon et al. (2002), we assumed the infection rate took the form

$$\phi_{S,N}=\alpha_{S,N}\frac{I}{N}S,$$

where $\alpha_{S,N}$is the product of per capita contact rate and the probability that an infection is successful, which was then multiplied by $\frac{I}{N}$, the probability that a contact is infectious, to estimate the force of infection. Note that $\alpha_{S,N}$is a function of S and N, and specifies the form of the transmission function $\phi_{S,N}$, which could take one of several functional forms (see below).

We used the optim function in R to fit eight candidate functional forms of $\phi_{S,N}$ to the infection status data simultaneously across the 20 mesocosms (Table 1) over the first 5 days of the experiment and ranked their suitability using Akaike's information criterion (AIC). Note that this required calculating the probability distribution of susceptible individuals separately for each treatment, using the ode function in R; all treatments were then incorporated into one maximum‐likelihood function representing the entire experiment (see supplementary information for details). The simplest candidate $\phi_{S,N}$ was density‐ and frequency‐dependent transmission (i.e. $\alpha_{S,N}=\beta N$ and $\alpha_{S,N}=\beta$, respectively, for some constant β; Table 2). We extended these functions to include saturating transmission at high densities, including the possibility that saturation was dependent on the population structure in terms of numbers of susceptible and infected individuals. We also considered power law dependence, which was a flexible but purely phenomenological model that provided a benchmark against which to compare our mechanistic formulations. Finally, we replaced the densities S and I=N‐S with the prevalences $x=\frac{S}{N}$ and $y=\frac{N‐S}{N}$ as an alternative form of population dependence (Table 2).

**TABLE 2:** Maximum‐likelihood parameter values for various forms of the transmission function $\phi_{S,N}$, listed in order by lowest AIC (i.e. best fit). Recall that N=S+I and $x=\frac{S}{N},\;y=\frac{I}{N}$. The lower limit in the optimization process was chosen as $10^{‐6}$ for all parameters, suggesting that any parameter reaching this value should be zero. FTC = ‘failed to converge’, indicating that the optimization process failed to find a minimum

Transmission type	Transmission function $\phi_{S,N}$	Parameters^a^	Negative log‐likelihood	AIC
Frequency‐dependent	$\frac{\beta SI}{N}$	β=0.230	63.2	128.3
Quadratic saturation	$\frac{\mathrm{SI}}{\kappa_{0}+\kappa_{1}N+\kappa_{2}N^{2}}$	$\kappa_{0}=0.026,\;\kappa_{1}=2.79,$ $\kappa_{2}=0.085$	62.2	130.3
Power law	$\beta S^{a}I^{b}$	β=0.176,a=0.362, b=0.382	63.2	132.4
Structural saturation	$\frac{\mathrm{SI}}{\kappa_{0}+\kappa_{S}S+\kappa_{I}I+\kappa_{2}SI}$	$\kappa_{0}=10^{‐6},\;\kappa_{S}=2.55,$ $\kappa_{I}=3.36,\;\kappa_{2}=0.366$	62.5	132.9
Simple prevalence	βxy	β=3.39	68.7	139.3
Power law (prevalence)	$\beta x^{a}y^{b}$	β=4.29,a=1.27, b=1.04	68.4	142.8
Quadratic saturation (prevalence)	$\frac{\mathrm{xy}}{\kappa_{0}+\kappa_{1}x+\kappa_{2}x^{2}}$	$\kappa_{0}=0.295,\;\kappa_{1}=10^{‐6}$ $\kappa_{2}=10^{‐6}$	68.7	143.3
Density‐dependent	βSI	FTC	FTC	FTC

^a^ The transmission function $\phi_{S,N}$has units time^‐1^. As S,IandN are numbers and xandy are prevalences, they are dimensionless quantities; thus, β also has units time^‐1^ throughout, and each of the κ parameters has unit time. a and b are dimensionless. $\kappa_{0}$ is equivalent to $\beta^{‐1}$, but is written in the denominator to allow the optimization algorithm to select $\kappa_{0}=0$.

### 2.6 Model validation

**FIGURE 1:**
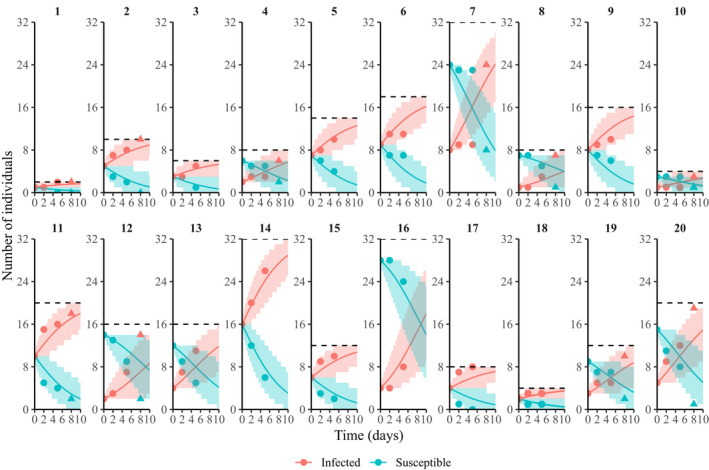
Data used to estimate transmission (circles), compared with the mean (solid lines) of the probability distribution of susceptible individuals as predicted by the best‐fitting model, frequency‐dependent transmission (Table 2). The model was fitted to data from days 0 to 5, the period with no host deaths or recoveries from infection. Triangles represent data from day 8 in the 10 mesocosms that had no deaths or recoveries; these were not used for model optimization but instead compared with model predictions as a form of validation. Minimum 95% confidence intervals for the data are represented by shaded regions. The dashed lines indicate the total population size of each treatment

The above model fitting was carried out over the first five days of the experiment, thus permitting us to neglect deaths and recoveries. For 10 out of the 20 mesocosms, no deaths had occurred by the next time point (day 8; Figure 1 and Figure S1). We did not include these data in the optimization in order to prevent those treatments having undue influence on the result; however, they provide one means of testing our model predictions. To this end, we calculated the negative log‐likelihood of the two best‐fitting models over days 5 to 8 for those mesocosms in which no deaths or recoveries had yet occurred, and compared the accuracy of their predictions using AIC.

### 2.7 Basic reproductive ratio, *R_0_*

Based on the best‐fitting model, combined with estimates from the literature, we derived an estimate of the basic reproductive ratio (*R_0_*) as follows. Assuming an SIR (susceptible–infectious–recovered) framework for *Bsal*, *R_0_* was defined as follows:

$$R_{0}=\frac{\alpha_{S,N}}{\left(\mu +\sigma \right)}$$

mode where $\alpha_{S,N}$ was defined as above in terms of numbers of individuals and assuming *S = N, µ*was the mortality rate, and *σ* was the recovery rate of infectious animals (Anderson & May, 1991). Malagon et al. (2020) reported an estimate of $\mu =0.0223\;d^{‐1}$ for eastern newts exposed to *Bsal* via host contact. In addition, infected eastern newts that do not die take on average 40 days to recover (D. Malagon, Clemson University, pers. comm.); so, $\sigma =\frac{1}{40}=0.025\;d^{‐1}$. We then combined these values with the best‐fit value of $\alpha_{S,N}$ from this study into the above equation to generate an estimate of *R_0_*.

## 3 RESULTS

### 3.1 Host infection and survival

Transmission occurred rapidly in mesocosms with >90% of susceptible individuals testing qPCR‐positive for *Bsal* within 17 days post‐exposure to an infected individual. All individuals in the experiment tested qPCR‐positive by 59 days post‐exposure. Total mortality among treatments at the end of the 62‐day experiment was 45%. We detected no differences in mortality rate among host density and infection prevalence treatments (Figure S1). *Bsal* load on the skin of infected hosts differed among host densities (coefficient for density effect on load = 0.013; *p = 0.03*) with individuals in higher density treatments experiencing greater average loads (Figure S2).

### 3.2 Modelling transmission

**FIGURE 2:**
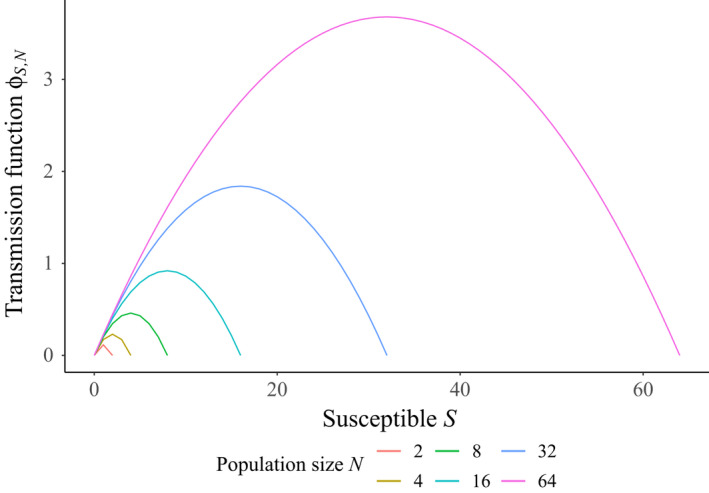
Frequency‐dependent transmission function ($\phi_{S,N}=\frac{\beta SI}{N}$) at the maximum‐likelihood fit β=0.230, plotted as a function of the density of susceptible hosts (*S*) for various total population sizes (*N*)

**FIGURE 3:**
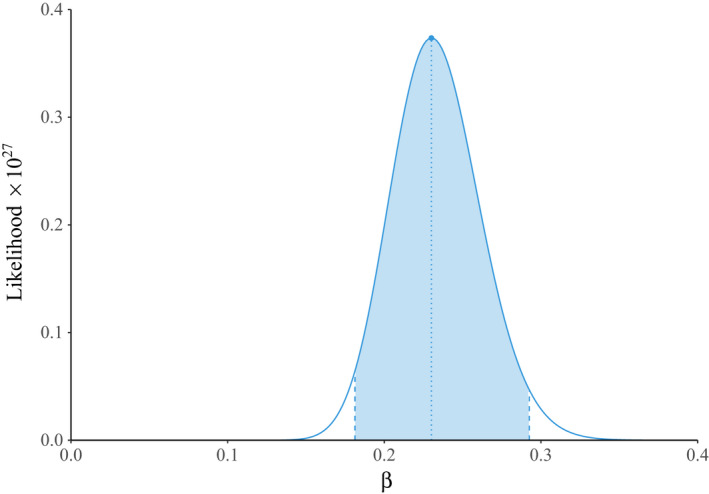
Likelihood profile for the parameter β in frequency‐dependent transmission. The location of the maximum likelihood is indicated by the dotted line and corresponds to the value β=0.230 given in Table 2. The shaded area represents 95% of the area under the curve, yielding the confidence interval β∈[0.181,0.293]

The results of our model fitting are summarized in Table 2. We found that the transmission function that minimized the AIC was pure frequency‐dependent transmission, suggesting that per capita contact rates $\alpha_{S,N}$ did not vary with host density (Begon et al. 2002), at least at the densities used in our experiment. Predictions of the best‐fitting model to the data from each treatment are presented in Figure 1, and the behaviour of this optimal $\phi_{S,N}$ in relation to the density of susceptible individuals (*S*) is plotted in Figure 2, demonstrating how the transmission function $\phi_{S,N}$ increases with total population size N. The likelihood profile for β in this case can be seen in Figure 3, with maximum likelihood at β=0.230 and 95% of the profile area lying in the range β∈[0.181,0.293].

The model with the next highest AIC corresponded to quadratic saturation (Table 2), meaning that the numerator in the contact function $\alpha_{S,N}$ is a quadratic function of N. Whereas frequency‐dependent transmission predicts a constant per capita contact rate, quadratic saturation implies that contact rate initially increases, peaks and then decreases with increasing population density. The most meaningful mathematical difference between the two functions was that with quadratic saturation, the peak of the transmission function $\phi_{S,N}$ was bounded above for large *N*, whereas the peak of frequency‐dependent transmission increased without bound in the same limit (Figures S3 and S4). It is worth noting that the negative log‐likelihood was lower for quadratic saturation than for frequency dependence, indicating that although the fit was better, the model was penalized for containing two additional parameters. Similarly, incorporating population structure into the saturation terms (denoted structural saturation in Table 2) did not sufficiently improve the model fit to overcome the penalty to the AIC for including more parameters.

### 3.3 Model validation

**FIGURE 4:**
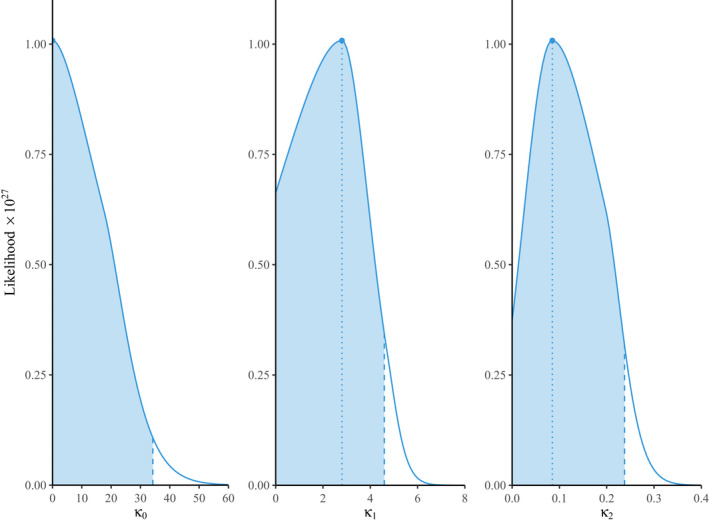
Likelihood profiles for the parameters in quadratically saturating transmission. The location of the maximum likelihood for each parameter is given by the dotted line and corresponds to the parameter values $\kappa_{0}=0.026$, $\kappa_{1}=2.79$ and $\kappa_{2}=0.085$ given in Table 2. The shaded areas represent 95% of the area under each curve, yielding the confidence intervals $\kappa_{0}\in \left[0,34.2\right]$, $\kappa_{1}\in \left[0,4.59\right]$ and $\kappa_{2}\in \left[0,0.238\right]$

The AIC for days 5 to 8 was 65.9 for the frequency‐dependent model, while for the quadratic saturation model it was 70.6, suggesting that frequency‐dependent transmission continued to be a better fit. Furthermore, we plotted the likelihood profiles for both models in Figures 3 and 4. In the case of the multi‐parameter quadratic saturation model, these were the trajectories through parameter space given by fixing one parameter at successive points along a given range and optimizing over the remaining two. These results suggest there was much greater uncertainty in the parameter values for the quadratic saturation model (Figure 4); in particular, all three parameter ranges began at zero. In contrast, the likelihood profile for the frequency‐dependent model was narrowly peaked away from zero (Figure 3), thus providing further evidence for its selection as the best‐fit model.

### 3.4 Basic reproductive ratio, *R*_0_

Using our frequency‐dependent transmission model ($\alpha_{S,N}=\beta$= 0.230) and previous estimates for host mortality and recovery rate (Malagon et al., 2020), we estimated *R*_0_ for *Bsal* to be 4.9 in an eastern newt population.

## 4 DISCUSSION

The best‐fit model for *Bsal* transmission between adult eastern newts in aquatic mesocosms was pure frequency‐dependent transmission. This is the first study that directly measured and modelled the transmission of *Bsal* in a host population. Previous studies either assumed density‐dependent transmission (Canessa et al., 2018; Schmidt et al., 2017) or measured contacts between uninfected hosts to infer transmission (Malagon et al., 2020). Our results add to the growing empirical recognition that transmission of wildlife pathogens can saturate and be functionally frequency‐dependent for all but the smallest host densities (Brunner et al., 2017; Brunner & Yarber, 2018; Hopkins et al., 2020). Several reasons might exist for frequency‐dependent transmission in the *Bsal*–eastern newt system. First, the probability of transmission via contact of an infected eastern newt with an uninfected susceptible individual is >95% (Malagon et al., 2020). Second, at our lower density treatments (i.e. 2–8 newts per m^2^), which are indicative of the wild, eastern newts contact each other between 90 and 1,000 times per day in aquatic mesocosms with and without habitat structure (Malagon et al., 2020). Hence, the likelihood of *Bsal* transmission is almost guaranteed regardless of host density, which is supported by our result that >90% of the susceptible newts tested positive for *Bsal* infection within 17 days post‐exposure to an infected newt. That said, if host density was decreased below our lowest treatment density (i.e. <2 newts per m^2^), it is possible that density‐dependent transmission could occur, and this relationship should be explored. Similarly, we found little evidence of an upper bound on transmission, as would be provided by, for example, a quadratically or structurally saturating transmission function. Although it is unlikely that the peak of the transmission function can actually increase without bound as population density increases, it may be that such a phenomenon only occurs at unfeasibly high host densities.

Interestingly, when the optimization process was applied to what is perhaps the simplest model, namely density‐dependent transmission, it failed to converge to a minimum. This appears to be due to overly rapid transmission in simulated populations with larger densities, resulting in all simulated individuals becoming infected too quickly to be consistent with the data. Note that all other functional forms of the transmission function $\phi_{S,N}$ incorporated a contact rate $\alpha_{S,N}$ that was either constant or concave‐down with increasing population density, a feature absent from density‐dependent transmission. This strongly suggests that, consistent with Malagon et al. (2020), contact rates decrease with higher host densities, slowing infection rates compared with density‐dependent transmission.

In addition to host density, Wilber et al. (2017) also reported that the zoospore pool played an important role in *Bd* transmission; however, transmission in our system was likely driven by host contacts because we used transmission data from the first five days of the experiment (i.e. prior to any host mortality); hence, it is unlikely that substantial zoospore shedding occurred. Islam et al. (2021) simulated *Bsal* transmission and zoospore shedding in eastern newts, and it took several days for zoospores to accumulate beyond an infectious‐dose concentration in stagnant systems. Further, our mesocosms were flow‐through with water turning over ca. 2X per day, which would presumably reduce transmission by free‐swimming zoospores and support that host contacts were the primary driver of transmission in our system.

We detected no differences in mortality rates among density treatments, but there was some evidence that *Bsal* load was positively related to host density. While this result was driven by hosts in the highest infection prevalence treatment, this provides some interesting evidence that at higher host density and infection prevalence the zoospore pool contributes more to transmission, facilitating increases in *Bsal* loads on the skin. Alternatively, high density also could increase host stress and facilitate *Bsal* growth. In *Bd‐*amphibian systems, it is often assumed that on‐host infection processes, such pathogen growth, decouple from transmission processes (DiRenzo et al., 2018; Wilber et al., 2017), but our results suggest that this assumption might require increased scrutiny in *Bsal* systems, with important implications for modelling *Bsal*–amphibian dynamics.

We estimated *R*_0_ = 4.9, which is somewhat higher than previously estimated by Malagon et al. (2020), who estimated *R*_0_ = 1.9–3.2, suggesting invasion probability of *Bsal* into adult eastern newt populations is high. Canessa et al. (2018) estimated *R*_0_ = 5.8–14.3 for European fire salamanders where no management actions occurred, which might reflect greater susceptibility of this species to *Bsal* infection. However, considering that many of the parameter estimates in their models were based on expert opinion rather than based on experimental data designed to infer transmission rates, it is possible their *R*_0_ was overestimated.

Theory predicts that pathogens that exhibit pure frequency‐dependent transmission are able to persist at very low host densities, and so have the potential to drive their host population extinct (McCallum et al., 2001). However, in reality, we would expect transmission to transition to density dependence for sufficiently small host densities (Antonovics et al., 1995). This would also be true if transmission occurred primarily through contact with zoospores in the environment at low host densities (Anderson & May, 1978), although robust populations of *Bsal* zoospores in the environment could effectively lead to frequency‐dependent transmission even at low host densities (Islam et al., 2021; Stegen et al., 2017). Although density‐dependent transmission would render deterministic extinction impossible, at very low densities stochastic extinctions become increasingly likely (Lloyd‐Smith et al., 2005). Hence, the epidemiological implications of frequency‐dependent transmission coupled with *R*_0_ > 1 suggest that disease management actions will likely need to be extreme to control an outbreak of *Bsal* in eastern newt populations, similar to fire salamanders (Canessa et al., 2018). Typically, best management strategies for reducing the population‐level effects of frequency‐dependent transmission focus on increasing host resistance or tolerance to infection or decreasing environmental persistence of the pathogen (Almberg et al., 2011; McCallum, 2008, 2012). Anti‐fungal or microbiome treatments that help hosts clear or tolerate *Bsal* infections have promise for success (Bletz et al., 2018; Blooi et al., 2015); however, widespread application in large host populations or across multiple sites will be logistically difficult (Canessa et al., 2018). Environmental application of fungicides or anti‐*Bsal* microbes might be one approach to large‐scale management, although effects on other organisms need to be considered (Silva et al., 2019; Woodhams et al., 2018). Canessa et al. (2018) suggested that reducing fire salamander density by 50%–90% could reduce *R*_0_, which our results do not support for eastern newts, although reduction in host densities below our lowest treatment (<2 individuals per m^2^) might be effective and should be explored.

Collectively, these results support the recommendations in European systems that management actions should focus on preventing the introduction of *Bsal* (Schmidt et al., 2017; Thomas et al., 2019). Currently, very few nations have regulations requiring pathogen‐free trade of amphibians (Grant et al., 2017). Additionally, we now know that anurans are capable of becoming infected with *Bsal* (Nguyen et al., 2017; Stegen et al., 2017), which comprise 99% of amphibian trade (Can et al., 2019). Given global amphibian trade generates at least $6B USD annually (Can et al., 2019; Smith et al.,  2017), we encourage nations to develop subsidy programmes that facilitate clean trade. This mitigation strategy is especially important for North American countries that comprise >50% of the global diversity of salamanders (Yap et al., 2015), which are at greatest threat to *Bsal* invasion (Gray et al., 2015).

## CONFLICT OF INTEREST

The authors declare that they have no conflict of interests.

## ETHICAL APPROVAL

The authors confirm that the ethical policies of the journal, as noted on the journal's author guidelines page, have been adhered to, and the appropriate ethical review committee (University of Tennessee Institutional Animal Care and Use Committee) approved all research described herein (Protocol #2623). All husbandry and euthanasia procedures followed recommendations provided by the American Veterinary Medical Association and the Association of Zoos and Aquariums.

## Supporting information

Supplementary Material

## Data Availability

The data that support the findings of this study are openly available at https://dx.doi.org/10.7290/xw8yy2RBeu
