# Supplementary material for: Frequency‐dependent transmission of Batrachochytrium salamandrivorans in eastern newts
Source: Transbound Emerg Dis. 2021 Mar 9;69(2):731–41. doi: 10.1111/tbed.14043 (PMC9290712; doi:10.1111/tbed.14043)
Supplement: Supplementary file 1 — Supplementary Material [file TBED-69-731-s001.pdf]

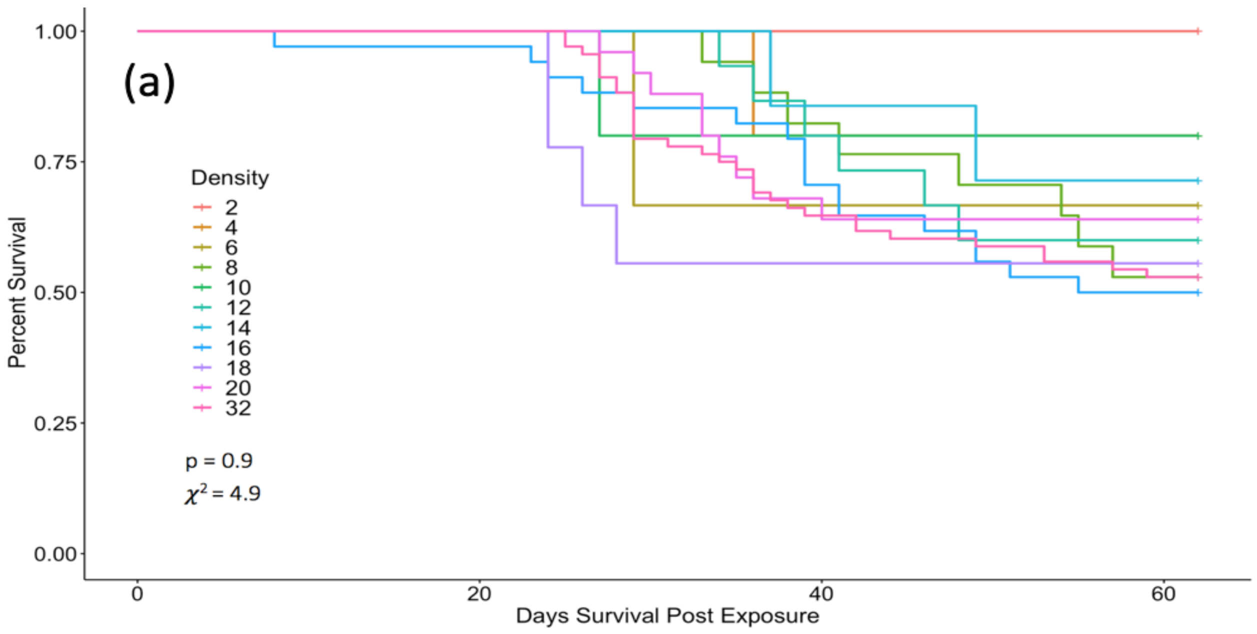

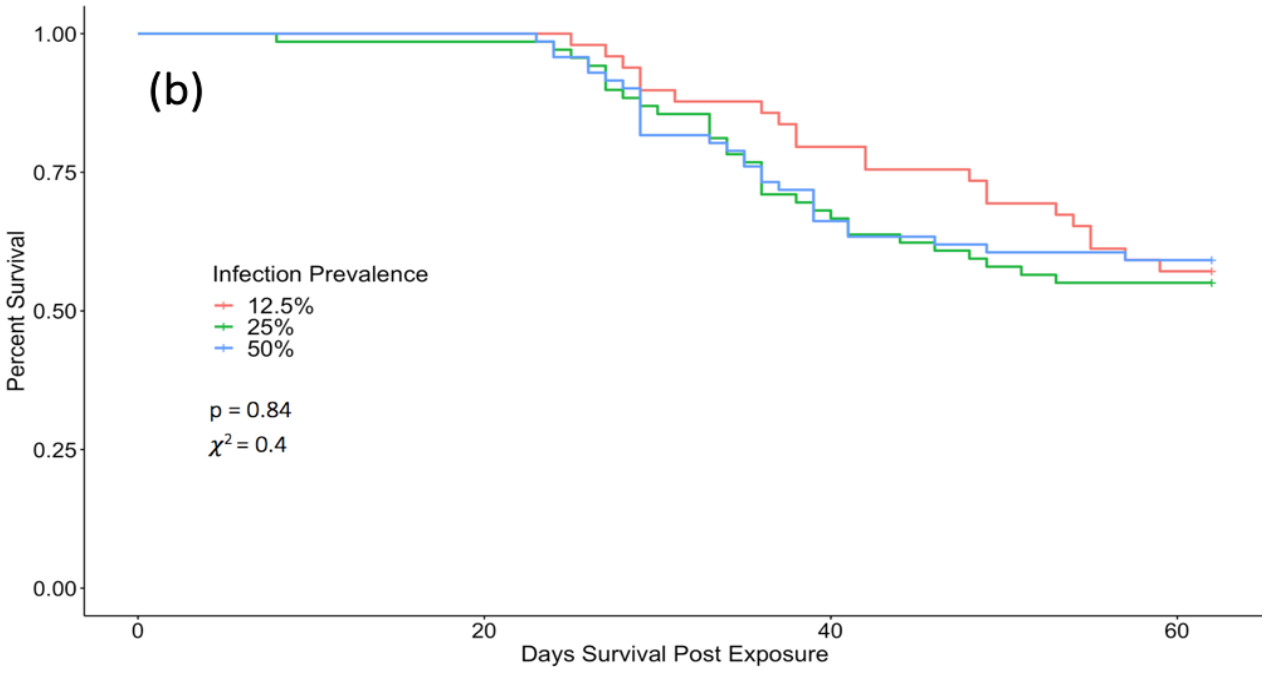

Figure S1: Kaplan-Meier survival analyses for susceptible hosts (*Notophthalmus viridescens*) following exposure to *Batrachochytrium salamandrivorans*-infected hosts at different host density (a) and infection prevalence (b) treatments.

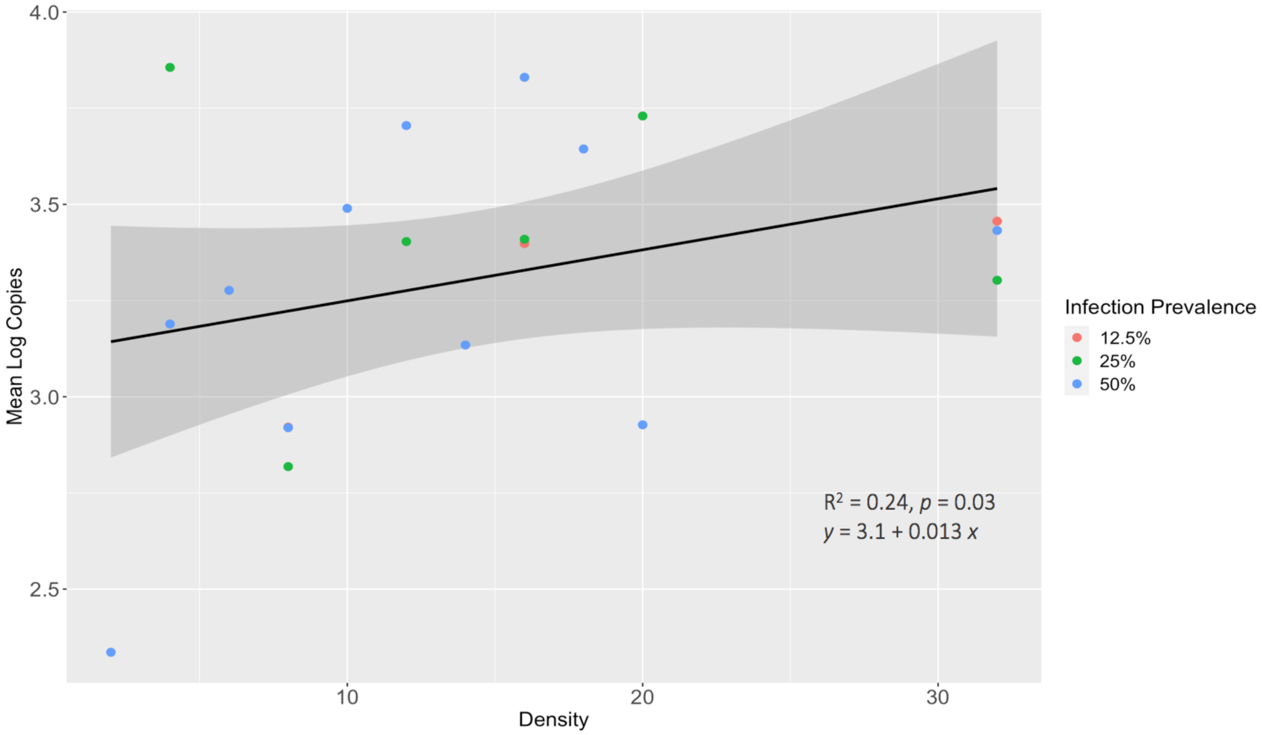

Figure S2: Average loads of *Batrachochytrium salamandrivorans* on *Notophthalmus viridescens* skin among host densities. The regression line (black line) and 95% confidence interval (gray shaded area) depict the linear relationship of host load (mean log *Bsal* copies/μL per tank) and host density per mesocosm. Points are colored by infection prevalence 12.5% (red), 25% (green) and 50% (blue). The R^2 value, coefficient and p-value reported are from the best fitting weighted regression model determined through model selection. Based on non-significant likelihood ratio tests for infection prevalence and the interaction of infection prevalence and density, we removed those terms from the model resulting in a final model with host density being the sole predictor of *Bsal* load.

Appendix: Maximum likelihood fitting of a probabilistic transmission model

1 Modelling transmission

The classical mathematical formulation of disease transmission is the deterministic SI model. Considering a large, well-mixed population of size N, we divide the population into uninfected (susceptible) and infected subpopulations, denoted respectively by S and I (so $S + I = N$), transmission dynamics can then be modelled by the ODE

$$\frac{\mathrm{d}S}{\mathrm{d}t} = \alpha \frac{I}{N} S = \alpha \left(1 - \frac{S}{N}\right) S. \tag{1}$$

Here α is the product of the per-capita contact rate and the probability an infection is successful, often simply referred to as the contact rate. Note that this ignores such processes as recovery and death, and that α is in general a function of S and N. Such an approach is invalid for small populations, wherein stochastic effects become relevant and a probabilistic approach is required.

To this end, consider a population of N individuals, with S_0 uninfected (susceptible) individuals and $I_0 = N - S_0$ infected at time $t = 0$. We define $p_{S|N,S_0}(t)$ to be the probability of S susceptible individuals at time t, given a total population N and initial susceptible subpopulation S_0. Assuming that death and recovery occur with negligible rates and can therefore be neglected over a sufficiently short time period, the distribution $p_{S|N,S_0}$ is determined by the S_0 differential equations

$$\frac{\mathrm{d}p_{S|N,S_0}}{\mathrm{d}t} = \phi_{S+1,N} p_{S+1|N,S_0} - \phi_{S,N} p_{S|N,S_0}, \quad 0 \leq S \leq S_0, \tag{2}$$

where $\phi_{S,N}$ is the transmission function, given S susceptible individuals (and hence $I = N - S$ infected). We suppose that $\phi_{S,N}$ takes the form

$$\phi_{S,N} = \alpha_{S,N} \left(1 - \frac{S}{N}\right) S, \tag{3}$$

where $\alpha_{S,N}$ is the contact rate for the particular host-pathogen pair, and set $\alpha_{S_0+1,N} \equiv 0$. In general, $\alpha_{S,N}$ also depends upon a parameter vector which we denote by θ. Note that if $S = 0$ or $S = N$ then infection events cannot occur. We emphasise that the probabilistic (2) and deterministic (1) formulations are equivalent only if each of N, S and I are considered to be numbers, not densities. To recast (1) in terms of densities, α and any parameters implicitly contained therein must be scaled appropriately by the system size.

We want to test different forms of the transmission function $\alpha_{S,N}$, with contacts either constant or increasing for small densities, and allowing for the possibility that the transmission function $\phi_{S,N}$ saturates with increasing population density. For this to hold for all values of $S = 0, \ldots, N$, in particular $S \sim N/2$, the transmission rate $\alpha_{S,N}$ must increase like N for $0 < N \ll 1$ and decay like $1/N^2$ as $N \to \infty$. Perhaps the simplest option is

$$\alpha_{S,N} = \frac{N}{\kappa_0 + \kappa_1 N + \kappa_2 N^2}, \tag{4}$$

which we term quadratic saturation. If $\kappa_1 = \kappa_2 = 0$, this yields density-dependent transmission; if $\kappa_0 = \kappa_2 = 0$ it is frequency-dependent; if only $\kappa_2 = 0$, it is density-dependent for low values of N and frequency-dependent for high values. However, in this case $\alpha_{S,N}$ depends only on N, i.e. is independent of the precise configuration

of susceptible and infected individuals. A formulation taking the subpopulation structure explicitly into account is

$$\alpha_{S,N} = \frac{N}{\kappa_0 + \kappa_S S + \kappa_I (N - S) + \kappa_2 S(N - S)}, \tag{5}$$

which we term structural saturation and does depend on the numbers of susceptible and infected (unless $\kappa_S = \kappa_I$ and $\kappa_2 = 0$; note that κ_2 multiplies a different quadratic term than in (4)). An alternative approach is to consider transmission to be a function of the prevalences of susceptible and infected, rather than the frequencies S and I; this yields

$$\alpha_{S,N} = \frac{1}{N} \frac{1}{\kappa_0 + \kappa_1 S/N + \kappa_2 (S/N)^2}. \tag{6}$$

Finally, we consider the simple power laws

$$\phi_{S,N} = \beta S^a (N - S)^b \tag{7}$$

and

$$\phi_{S,N} = \beta (S/N)^a (1 - S/N)^b; \tag{8}$$

although these do not saturate, in both cases $\alpha_{S,N}$ does decrease with increasing N provided $a + b < 1$. Setting $a = b = 1$ in (7) yields simple density-dependent transmission, equivalent to (4) with $\kappa_1 = \kappa_2 = 0$, while doing so in (8) is equivalent to (4) with $\kappa_0 = \kappa_1 = 0$.

2 Parameter fitting

Our experiment consists of M treatments, each of which is characterised by the total population N_m and initial susceptible subpopulation $S_{m,0}$, for $m = 1, \ldots M$. Each treatment yields a time series $S_{m,0:K}$ of K data points detailing the number $S_{m,k}$ of susceptible individuals at times $t = t_k$, $k = 0, 1, \ldots, K$. These data are plotted in Figure 1 of the main text. The negative log-likelihood $\mathrm{NLL}(\theta|N, S_{0:K})$ of a parameter choice θ given a population size N and a time series $S_{0:K}$ is therefore

$$\mathrm{nll}(\theta|N, S_{0:K}) = -\sum_{k=1}^{K} \log(p_{S_k|N,S_0}(t_k)). \tag{9}$$

The probabilities $p_{S_k|N,S_0}(t_k)$ are readily calculated by numerical integration of (2) with the appropriate choice of $\alpha_{S,N}$. The negative log-likelihood $\mathrm{NLL}(\theta)$ of the whole experiment is then given by summing (9) over all M treatments, i.e.

$$\mathrm{NLL}(\theta) = \sum_{m=1}^{M} \mathrm{nll}(\theta|N_m, S_{m,0:K}). \tag{10}$$

Thus, minimising (10) with respect to θ yields the parameter values of maximum likelihood.

We used the `optim` function in R to minimise (10) for each of the transmission functions (4)-(8), including the special cases (4) with $\kappa_0 = \kappa_2 = 0$ and both (7) and (8) with $a = b = 1$. The results are summarised in Table 2 in the main text. The different choices of transmission function are plotted in Figures S3 and S4, using the optimal parameter values of Table 2.

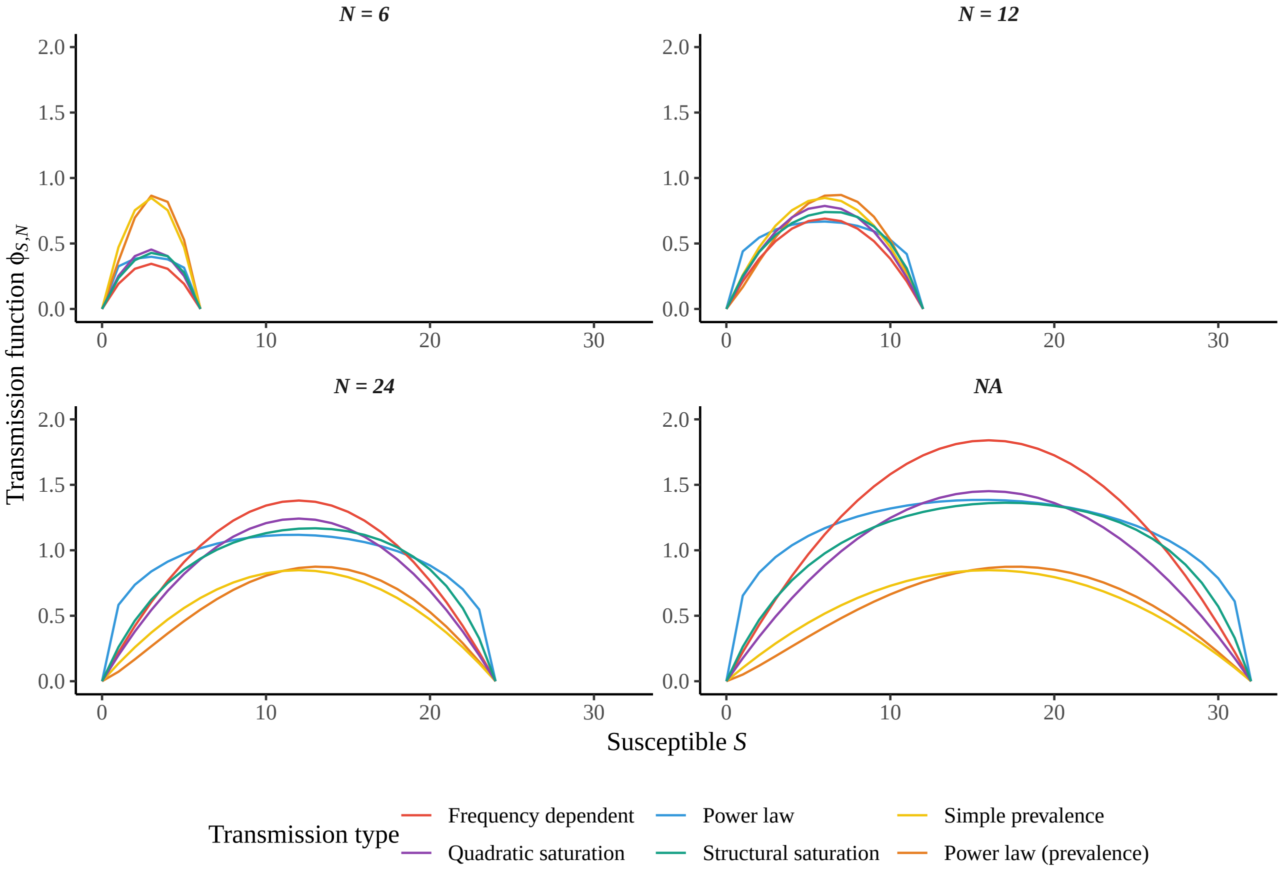

Figure S3: Plots of the different choices of the transmission function $\phi_{S,N}$ for four values of N. Parameter values are as given in Table 2. Quadratic saturation as a function of prevalence is omitted, as the best fit for this transmission type was equivalent to simple prevalence.

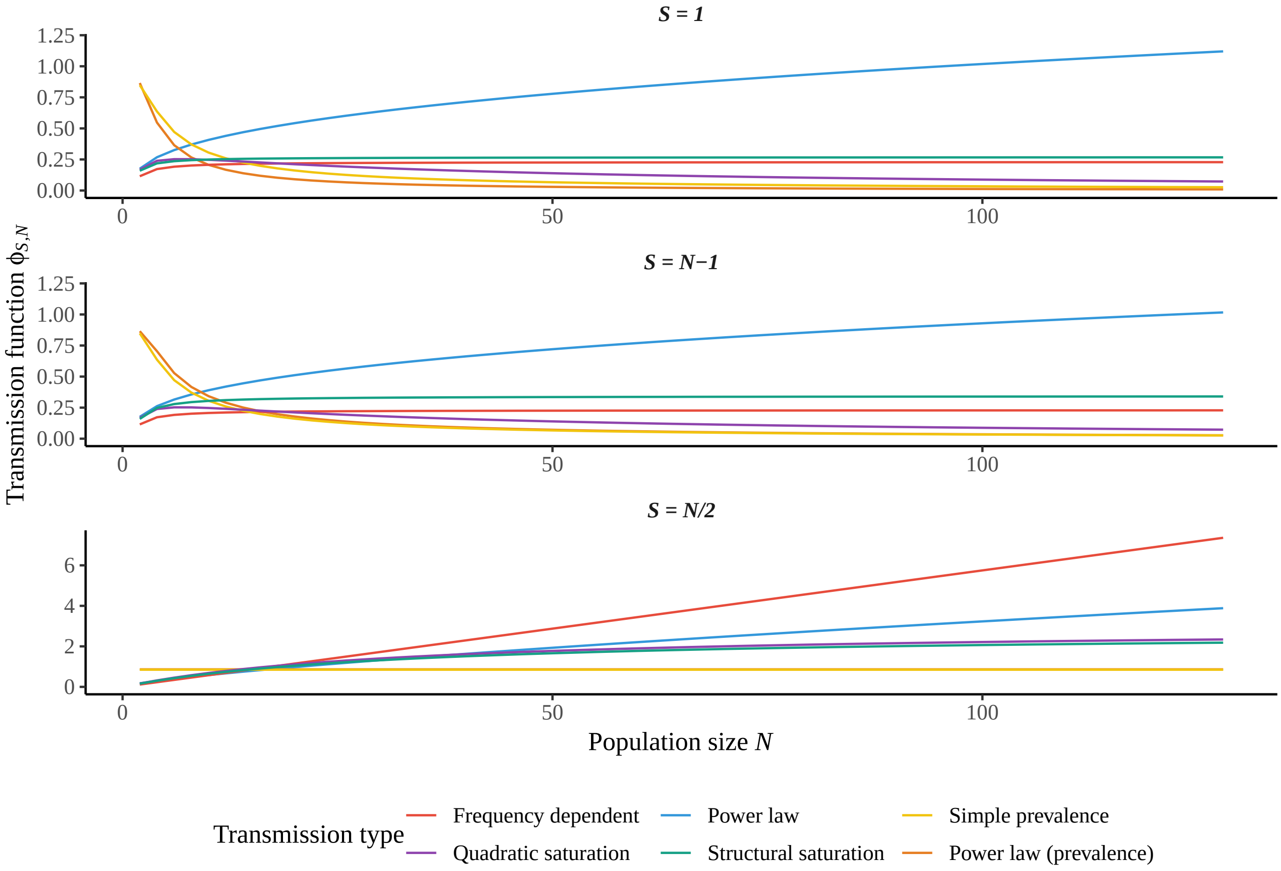

Figure S4: Plots of the different choices of the transmission function $\phi_{S,N}$, evaluated at $S = 1, N-1, N/2$ for increasing values of N. Parameter values are as given in Table 2. Quadratic saturation as a function of prevalence is omitted, as the best fit for this transmission type was equivalent to simple prevalence.
